# Raw Milk Cheese Microbiomes: A Paradigm for Interactions of Lactic Acid Bacteria in Food Ecosystems

**DOI:** 10.3390/foods15071160

**Published:** 2026-03-30

**Authors:** Christine K. Olupot, Olivia Sheehan, Zoe Kampff, Brian McDonnell, David F. Woods, Gabriele Andrea Lugli, Marco Ventura, F. Jerry Reen, Douwe van Sinderen, Jennifer Mahony

**Affiliations:** 1 School of Microbiology, University College Cork, T12 YT20 Cork, Ireland; 119221114@umail.ucc.ie (C.K.O.); 121348006@umail.ucc.ie (O.S.); 115356676@umail.ucc.ie (Z.K.); brian.mcdonnell@ucc.ie (B.M.); david.woods@ucc.ie (D.F.W.); j.reen@ucc.ie (F.J.R.); d.vansinderen@ucc.ie (D.v.S.); 2 APC Microbiome Ireland, University College Cork, T12 YT20 Cork, Ireland; 3 Laboratory of Probiogenomics, Department of Chemistry, Life Sciences and Environmental Sustainability, University of Parma, 43124 Parma, Italy; gabrieleandrea.lugli@unipr.it (G.A.L.); marco.ventura@unipr.it (M.V.); 4 Microbiome Research Hub, University of Parma, 43124 Parma, Italy; 5 SSPC, The Research Ireland Centre for Pharmaceuticals, University College Cork, T12 YT20 Cork, Ireland

**Keywords:** food fermentations, metagenomics, culture-dependent analysis, starter lactic acid bacteria, non-starter lactic acid bacteria, *Lactococcus*, *Streptococcus*, *Hafnia* spp.

## Abstract

While industrial-scale dairy fermentations often employ pasteurized milk as the substrate, many farmhouse and traditional production practices apply raw milk derived from a variety of mammals. Certain artisanal production systems rely on the autochthonous microbiota of the milk, fermentation vessels, equipment and/or environment to initiate milk coagulation. While the technological properties of lactic acid bacteria associated with dairy fermentations are well described, their interactions with other organisms during fermentation and cheese ripening are poorly investigated. This study presents an overview of the microbial ecology of raw and pasteurized milk used in the production of Irish farmhouse cheeses using metagenomic and culture-based approaches. Metagenomic analysis of four raw milk-derived cheeses established the dominant presence of either lactococci or *Streptococcus* spp. and with a secondary population of various lactobacilli. Interestingly, the Brie sample was also demonstrated to possess significant proportion of *Hafnia* spp. This was corroborated in culture-based analysis where *Hafnia* isolates were also identified. Furthermore, we report on the motility phenotype, lactose utilization ability and metabolic products of isolates of *Hafnia paralvei* and *Hafnia alvei*, and determine that these strains could grow in a non-antagonistic manner on plates with strains of *Lactococcus lactis* and *Streptococcus thermophilus*. As artisanal and farmhouse production systems are often associated with protected or regionally significant products, it is essential to develop a clear understanding of the microbial communities within and the complex relationships between the community members.

## 1. Introduction

The dairy sector is a major contributor to the Irish economy. Ireland is the third highest producer of cheese, average per person globally (56.7 kg per person) with almost 300,000 metric tonnes of cheese produced each year. While Ireland is renowned for its production of Cheddar-style cheeses using so-called “defined” bacterial starter cultures [1,2,3], there is considerable growth in farmhouse cheese manufacture which uses artisanal production processes and undefined starter cultures [4]. Therefore, the range of cheeses now being produced in Ireland includes soft cheeses such as Brie and Camembert, semi-soft cheeses including Mozzarella and Reblochon and hard cheeses including Smoked Drumlin. While many of these cheese types have international origins, the names of the cheeses refer to the style of cheese rather than a specific protected designation of origin (PDO) cheese, e.g., Brie de Meaux. Artisanal production systems often involve the use of raw, unpasteurized bovine or ovine milk as substrates for fermentation. While these foods are considered safe for human consumption, detailed knowledge of the microbiota of such products is limited. Furthermore, while the majority of bacteria that contribute to these fermentations are likely to be members of the heterogenous group of lactic acid bacteria (LAB) including *Lactococcus*, *Streptococcus* and lactobacilli, the microbiota of raw milk cheeses often contain enteric organisms including members of the *Enterobacteriaceae* and non-starter LAB, such as *Enterococcus* spp. [5]. These species may contribute to the flavor profile of the product, yet may simultaneously contribute to the dissemination of antibiotic-resistance associated genes within the human gastrointestinal microbiota, which is a major public health concern [6,7].

Certain LAB have long been applied deliberately in the production of fermented dairy foods [8,9]. Furthermore, species such as *S. thermophilus* and *L. lactis* have for a long time have been safely used in fermented foods and are widely employed in industrial food production. In the Unites States, the Food and Drug Administration (FDA) establishes safety assessment through the GRAS (Generally Recognized as Safe) framework to evaluate particular strains and their use [10,11]. Similarly, EFSA (European Food Safety Authority) applies QPS (Qualified Presumption of Safety) to strains following a safety evaluation [12].

Sustainability in dairy production systems is beneficial for the continued success and further growth of the agri-food sector. With growth in farmhouse cheese production sites and volumes in Ireland, it is becoming increasingly important to define the microbial communities that underpin such artisanal fermentations. The role of LAB in acidifying milk, ripening and flavor formation and rapid completion of the fermentation is essential in raw milk-based fermentations since slow or incomplete fermentations result in nutrients becoming available to non-LAB spoilage or pathogenic organisms [13]. Strains of the Gram-negative bacterium *Hafnia* are commonly isolated from raw milk and (particularly) soft cheeses [14,15,16,17]. However, while this bacterium has been reported as a contributor to good quality cheese in the context of ripening and flavor development [11,12], it may also be associated with negative outcomes particularly if the overall abundance of *Hafnia* is high [13].

While several studies have evaluated the presence of specific bacterial species [5,13,18,19] and while there are emerging reports of the metagenomic evaluation of fermented foods [20,21,22,23], there are limited reports that provide a holistic understanding of the microbiota of artisanally produced cheeses and particularly those produced in an Irish context [18,24,25]. Therefore, the current study aimed to define the microbiota of Irish farmhouse, artisanally produced cheeses using culture-dependent and independent approaches. Strains of *Hafnia* spp. were isolated from samples of Brie, Camembert and Reblochon cheeses, which were characterized to establish their impact and possible role in such products. Finally, we sought to explore the interactions between lactic acid starter bacterial isolates and non-LAB *Hafnia* isolates from these cheeses.

## 2. Materials and Methods

### 2.1. Cheeses Evaluated in This Study

Twelve cheeses were analyzed in this study including Brie (n = 2), Camembert (n = 2) and Pecorino (n = 2) cheese samples as well as one Reblochon, Smoked Drumlin, Caciocavallo, Mozzarella, Saint Felicien and Fleur du Maquis cheese samples. All twelve cheeses were sourced locally and produced in farmhouse production facilities in Cork, Ireland in 2024. Six of the cheese samples representing five cheese types (Fleur de Maquis, Saint Felicien, Mozzarella, Caciocavallo and Pecorino) were produced using pasteurized milk and six cheeses representing four cheese types (Brie, Camembert, Smoked Drumlin and Reblochon) were produced from raw milk (Table 1). Four different “young” raw bovine milk-derived cheeses (fresh and briefly aged, 1 week to 3 weeks old) were selected for metagenome sequencing based on having distinct textural properties and production conditions (e.g., Camembert, Smoked Drumlin and Brie are typically produced at mesophilic temperatures while Reblochon is produced at the higher end of the mesophilic range and is reported to incorporate both mesophilic and thermophilic organisms) [26].

### 2.2. Metagenomic DNA Extraction

DNA extractions were performed for the above-mentioned raw milk cheese samples as follows: 2 g samples from the core and rind/surface of each cheese wheel were aseptically collected using a pre-sterilized knife for DNA extraction. Each cheese sample was mixed with 25 mL of 2.2% sodium citrate (heated to 45 °C; Sigma-Aldrich St. Louis and Burlington, MA, USA) and homogenized in a stomacher 400 (Seward, Worthing, West Sussex U.K.) for 5 min at 230 rpm. The homogenized samples were centrifuged at room temperature (~20 °C) 13,800× *g* for 10 min after which the fat layers from the samples were removed. The supernatant from each sample was discarded, and the cells/cheese debris resuspended and washed in 1 mL of 2% sodium citrate (heated to 45 °C; Sigma-Aldrich). The sample was then centrifuged at 10,800× *g* for 5 min. The washing step was repeated three times. The washed cells were resuspended in 1 mL of lysis buffer (20 mM Tris HCl–pH 8, 2 mM EDTA- pH 8, 2% polyethylene glycol), 50 µg/mL lysozyme solution and 100 U mutanolysin (Sigma-Aldrich).

The solution was incubated at 37 °C for 3 h. After incubation, 250 µg/mL of Proteinase K was added to the solution and incubated again at 56 °C for 1 h. Subsequently, each solution was precipitated using 1 mL 96% ice cold ethanol. Using GenElute™ Bacterial Genomic DNA kit (Sigma-Aldrich), washing and elution of DNA was carried out according to manufacturer’s instructions, but applying 40 µL of elution buffer instead of 200 µL. A second elution was performed for each sample. The extracted DNA was quantified using Qubit™ dsDNA HS assay kit (Thermo Fischer Scientific, Waltham, MA, USA) and a Qubit^®^ 2.0 Fluorometer and visualized on a 1% agarose gel. Following this process, the DNA of four of the raw milk cheeses including a Brie, Camembert, Smoked Drumlin and Reblochon sample was selected for metagenomic analysis based on having the highest DNA yields and quality as representatives of the four raw milk-derived cheeses.

### 2.3. Metagenome Sequencing & Analysis

Using the extracted DNA, partial 16S rRNA gene sequences were amplified. Primer pairs Probio_Uni/Probio_Rev (CCTACGGGRSGCAGCAG/ATTACCGCGGCTGCT) were used, targeting the V3 region of the 16S rRNA gene sequence [27]. Overhang sequences by Illumina adapter were added, and library preparation was performed according to the 16S rRNA Metagenomic Sequencing Library Preparation Protocol (Part #15044223 Rev. B–Illumina).

Amplicon quality was assessed by electrophoresis, and purification was performed using a magnetic bead-based clean-up to remove primer dimers. DNA concentrations were quantified fluorometrically and normalized to 4 nM prior to pooling. Sequencing was performed using the Illumina NextSeq 2000 platform. Raw paired-end reads were processed with a custom QIIME2-based pipeline [20,28]. Reads were filtered to retain sequences longer than 140 bp, with an average quality score >20. Sequences containing homopolymers >7 bp or primer mismatches were excluded. Amplicon Sequence Variants (ASVs) were inferred using DADA2 [29] with 100% sequence homology. ASVs not observed at least twice in the same sample were removed. Taxonomic classification was performed with QIIME2 [20,28] using the SILVA reference database [30].

Alpha diversity was calculated using Observed ASVs, Chao1, and Shannon indices; beta diversity was assessed using weighted UniFrac distances and visualized via Principal Coordinates Analysis (PCoA).

### 2.4. Culture-Based Analysis

Culture-based analysis of the four cheese samples for which metagenomic profiling was also performed (Brie, Camembert, Reblochon, Smoked Drumlin) was undertaken by establishing the viable plate counts of total bacteria on tryptic soy agar (TSA; Sigma-Aldrich, USA); coliforms on MacConkey agar (Sigma-Aldrich); lactobacilli, lactococci and *Leuconostoc* spp. on de Man–Rogosa–Sharpe agar (MRS—Oxoid Ltd., Basingstoke, UK); thermophilic coccoid lactic acid bacteria on *S. thermophilus* isolation agar (HiMedia Ltd., Mumbai, India). The culture-based analysis of these four cheeses on LAB-enrichment media appeared to generate significant counts of potential non-LAB members [31,32]. Therefore, to ascertain if this selection of LAB counts could be refined for cheeses, eight additional cheese samples were evaluated using culture-based approaches alone (Table 1). For the remaining eight cheese samples, the culture-based analysis was confined to presumptive LAB counts on *S. thermophilus* agar and M17 agar (Sigma-Aldrich) supplemented with 0.5% lactose (LM17 agar) at 42 °C to selectively enrich for and to provide more accurate counts of the LAB population in the cheeses [32].

Five g of each cheese was aseptically transferred into a sterile stomacher bag using a sterile spatula. Additionally, 45 mL of ¼ Ringer’s solution (Merck, Darmstadt, Germany) was added to the cheese and homogenized for one minute using a stomacher (Stomacher Circular 400; Seward, UK). Serial dilutions (10^−1^ to 10^−4^) of the cheese homogenates were then prepared in ¼ strength Ringer’s solution. One hundred µL of each dilution sample was spread plated on respective agar plates with different media per selection and incubated overnight anaerobically or aerobically at 30 °C, 37 °C and 42 °C for the selection of bacterial isolates.

Viable counts were recorded after 24 h except for counts of isolates on MRS agar plates which were recorded after 48 h incubation. Single representative colony isolates displaying a morphology consistent with the dominant colony type were streaked on LM17 agar (or other media on which the organisms were originally isolated) to purify the isolates prior to glycerol stock preparation (Fisher Co., Loughborough, Leicestershire, UK) and storage at −70 °C.

### 2.5. Species Identification of Bacterial Isolates Using 16S rRNA Gene Sequencing

Bacterial isolates were maintained using the same medium and incubation conditions used for their isolation. Colonies of the isolates were used as template for 16S rRNA gene amplification. For the Polymerase Chain Reaction (PCR), Luc*fw* (tgcctaatacatgcaagt) and Luc*Rv* (cttgttacgacttcaccc) primers (which amplify the entire gene) [33], one *taq* polymerase master mix (BioLabs) and nuclease-free water (Thermo Scientific) were mixed and amplified under conditions of 94 °C for 3 min, followed by 30 cycles of 94 °C for 30 s, 50 °C for 30 s and 68 °C for 1 min and 30 s, and a final extension of 68 °C for 7 min [31]. The amplicons were purified using GenElute PCR Clean-up Kit (Sigma Aldrich) according to manufacturer’s instructions.

Gel electrophoresis was carried out on a 1% agarose gel diluted in Tris-Acetate-EDTA (TAE) buffer with 2.5 µL SYBR Safe DNA gel stain (RayBiotech, Peachtree Cors, GA, USA) added [24]. The amplicons were visualized using UV transillumination. PCR products were purified according to the PureLink^®^ PCR Purification Kit (Invitrogen, Carlsbad, CA, USA) manufacturer’s instructions. Sanger sequencing of PCR products was performed by Genewiz Inc. (Leipzig, Germany). Results from the generated sequences were analyzed using BLASTN analysis against available sequence data on National Centre for Biotechnology Information (NCBI) database “(https://blast.ncbi.nlm.nih.gov/Blast.cgi accessed 5 February 2025)”.

### 2.6. Characterization of Hafnia Isolates

#### 2.6.1. Gas Production Evaluation

Twelve 1 mL sterile durum tubes were each placed in sterile test tubes containing 10 mL LM17 broth. These were then inoculated with 100 µL of fresh overnight cultures of each of the isolated *Hafnia* strains (strains were named CO1 to CO12) and incubated overnight at different temperatures of 4 °C, room temperature (RT), 30 °C, 37 °C and 42 °C to establish if the strains produce gas. All tests were performed in triplicate.

#### 2.6.2. Organic Acid Production

*Hafnia* isolates were grown overnight in LM17 broth, and the culture was then centrifuged at 1409× *g* for 10 min. The resulting supernatant was filter-sterilized twice using 0.22 µM filters (Sarstedt, Nümbrecht, Germany). Seven hundred and fifty µL of each filtered supernatant was transferred in triplicate into pre-labeled HPLC vials. Seven hundred and fifty µL of uninoculated LM17 broth was used as a negative control for the assay. The supernatants of three independent biological replicates of each strain were analyzed. The organic acids were profiled and quantified using HPLC (Agilent, Santa Clara, CA, USA) coupled with a Refractive Index Detector (Agilent) [31]. The mobile phase used was HPLC grade Water (Merck, Germany) supplemented with 0.01N sulphuric acid (Merck). Separation of the organic acids into their profiled range was achieved on a Rezex ROA-Organic Acid H+ (8%), LC Column 300 × 7.8 mM with a flow rate of 0.6 mL/min. The column temperature was maintained at 65 °C, and the injection volume of the samples was 20 µL. Quantification of the organic acids of interest was completed using the OpenLab Chromatography Data Systems (CDS) Software version 2.8 (Agilent, Waldbronn, Germany). Graphpad prism software version 10.6.1 (892) was used to calculate the average and standard deviation of the triplicate data sets and to generate the associated graph. The *Hafnia* strains were also streaked on MacConkey agar to establish if they utilize lactose since they were isolated on media specific for LAB.

#### 2.6.3. Growth Temperature Range Evaluation

Overnight cultures of *Hafnia* strains were prepared in both LM17 and LB broth and incubated at 4 °C, room temperature (RT ~ 20 °C), 30 °C, 37 °C and 42 °C. Fresh overnight cultures were diluted in Ringer’s solution and plated on LM17 and LB agar for each of the following temperatures: 42 °C, 37 °C, 30 °C, 4 °C and at Room Temperature (RT ~ 20 °C) [34]. Plates were incubated overnight at the above-mentioned temperatures. Viable counts and colony morphologies were recorded after 24 h incubation. All assays were performed in triplicate. Graphpad prism software version 10.6.1 (892) was used to calculate the average and standard deviation of the triplicate data sets and to generate the associated graph.

Simultaneously, 100 µL of a fresh culture of the twelve *Hafnia* strains were inoculated into 10 mL LM17 broth and incubated over a 24 h period at 4 °C, Room Temperature (RT ~ 20 °C), 30 °C, 37 °C and 42 °C. OD600 nm readings at each temperature were recorded every two hours for the first 8 h and then a final reading was taken at 24 h. All assays were performed in triplicate.

#### 2.6.4. Salt Tolerance

LB broth was prepared with a final concentration of 3, 4 and 5% NaCl, respectively [34]. Tubes containing 10 mL of each of the salt-containing LB or LM17 broth were inoculated with 100 µL of fresh overnight cultures of the twelve *Hafnia* isolates and were then incubated for 24 h at 37 °C, after which the optical density at 600 nm (OD600) was recorded. The salt tolerance tests were performed in triplicate and analyzed in GraphPad prism software as described above.

#### 2.6.5. Microscopic Evaluation

The Gram staining technique using crystal violet, Gram’s iodine, alcohol and safranin was performed to evaluate the morphology of the *Hafnia* cells to evaluate aggregation activity and any differences imposed by growth in LM17 and LB growth media. A single colony from each strain grown in LM17 or LB agar was picked with a 1 µL loop and placed on a glass slide with a drop of water prior to staining using the standard Gram staining approach with crystal violet for 1 min, followed by a 1 min iodine treatment, decolorization with alcohol prior to counter-staining with safranin. Samples were imaged on a light microscope at 40× magnification (Supplementary Figure S1).

#### 2.6.6. Motility Assays

Motility assays for the *Hafnia* strains were performed using both LB and LM17 media. Fifteen mL of 1.2% technical agar was used as base agar for all motility assay plates. The agar base was overlaid with 0.3% LB or LM17 soft agar, respectively. Using filter tips, 2 µL of fresh culture of each *Hafnia* isolate (emanating from an overnight culture grown on the same medium) was carefully applied to the center of the soft agar. The plates were then incubated upright at 37 °C, and the plates were visually inspected, and phenotypes recorded at 24 and 48 h.

Eiken agar motility assays were also performed to assess swarming and swimming phenotypes for each of the twelve *Hafnia* isolates [35]. A solution of 0.8% Eiken broth and 0.6% Eiken agar (Eiken Chemical Ltd., Tokyo, Japan) with 0.5% glucose was prepared and to which NaCl was added at a final concentration of 3, 4 and 5% to establish the impact of the added salt/osmotic pressure on motility in comparison to a negative control without added salt. Using sterile 1 µL loops, a single colony of each freshly grown *Hafnia* strain was transferred from overnight LM17 agar plates. A single colony of each strain was tapped in the center onto the top layer of the Eiken agar. The plates were incubated upright on a flat tray without agitation at 37 °C. The strains were visually inspected after 24 h, and the phenotypes were recorded [35]. All assays were performed as independent biological triplicate assays.

#### 2.6.7. Hafnia and LAB Interaction Assays

Interaction assays were performed using an adapted version of a previously described plating technique [36]. Ten µL of fresh overnight cultures of either *S. thermophilus* CO-St16 or *L. lactis* LL1 and *H. paralvei* CO12 was spotted on two LM17 agar plates respectively. The LAB cultures were each spotted 0.5 cm apart from the *Hafnia* cultures and incubated overnight for 24 h. One agar plate spotted with *L. lactis*, and *H. paralvei* was incubated aerobically at 30 °C for one day to enable the *L. lactis* strain to grow optimally. *S. thermophilus* and the *H. paralvei* strains were incubated anaerobically at 42 °C to accommodate CO-St16. Plates were removed and incubated at Room Temperature (RT ~ 20 °C) for five days. Interactions between the two LAB isolates and the *Hafnia* isolates were observed for growth, compatibility and/or antagonism over a period of one week.

#### 2.6.8. Statistical Evaluation and Data

All biological assays were performed in triplicate, and the average and standard deviation were calculated by GraphPad prism software version 10.6.1 (892) for creating all graphs. Statistical comparisons of milk acidification biological triplicate results were performed using one-way ANOVA followed by Tukey’s post hoc test using GraphPad prism.

## 3. Results

### 3.1. Metagenomic and Culture-Based Analysis of Four Raw-Milk-Derived Cheeses

While artisanal and traditionally produced cheeses from various geographical regions are increasingly well studied, the microbiota of artisanally produced cheeses in Ireland (using milk derived from grass-fed cows in Ireland) has not been explored to date. Therefore, we aimed to define the microbiota of four such cheeses (Brie, Camembert, Smoked Drumlin and Reblochon) produced in Ireland using culture-based analysis and metagenomic analysis. To establish the total viable bacterial counts (on TSA) and the subpopulations of LAB (LM17 & MRS agar) and coliforms (MacConkey agar), four raw milk cheese samples were evaluated by cultivation at 30 °C and 37 °C. Total counts ranged between 10^6^ and 10^7^ cfu/mL (Table 2). Presumed LAB counts were observed to constitute the majority of the total population with high counts (~10^5^–10^6^ cfu/mL) being observed for three of the four cheese samples (the exception being Smoked Drumlin where there were no detectable coliforms). The numbers of culturable LAB were similar across all four samples (~10^6^–10^7^ cfu/mL) (Table 2).

To establish the dominant culturable species across the four assessed cheeses, 16S rRNA gene sequencing was performed for 17 presumptive LAB (Brie n = 5; Smoked Drumlin n = 4; Camembert n = 4; Reblochon n = 4) and six isolates from the total counts on TSA (Brie n = 2; Smoked Drumlin n = 2; Reblochon n = 2).

Among the presumptive LAB isolates derived from counts on LM17 agar, only three isolates were validated as LAB (one *Enterococcus* spp., one *Leuconostoc* (*Lc*) *mesenteroides* and one *Lactococcus* (*L.*) *lactis* isolate), while the remaining five isolates were represented by *H. alvei* (n = 2), *H. paralvei* (n = 1), *Staphylococcus equorum* (n = 2). With the exception of one isolate, the selected isolates from MRS agar (n = 9 total) were identified as LAB members, i.e., five *L. lactis* isolates (from Brie n = 3, Camembert n = 1 and Reblochon n = 1), one *Lactiplantibacillus plantarum* isolate (from Smoked Drumlin), one *Levilactobacillus brevis* (from Camembert) and one *Lc. mesenteroides* (from Reblochon).

The sole non-LAB isolate was identified as *Staphylococcus casei*. Among the six isolates derived from counts on TSA, *H. alvei* and *H. paralvei* were dominant (n = 2 and n = 3, respectively) while one *Enterobacter* spp. isolate was also identified.

**Figure 1:**
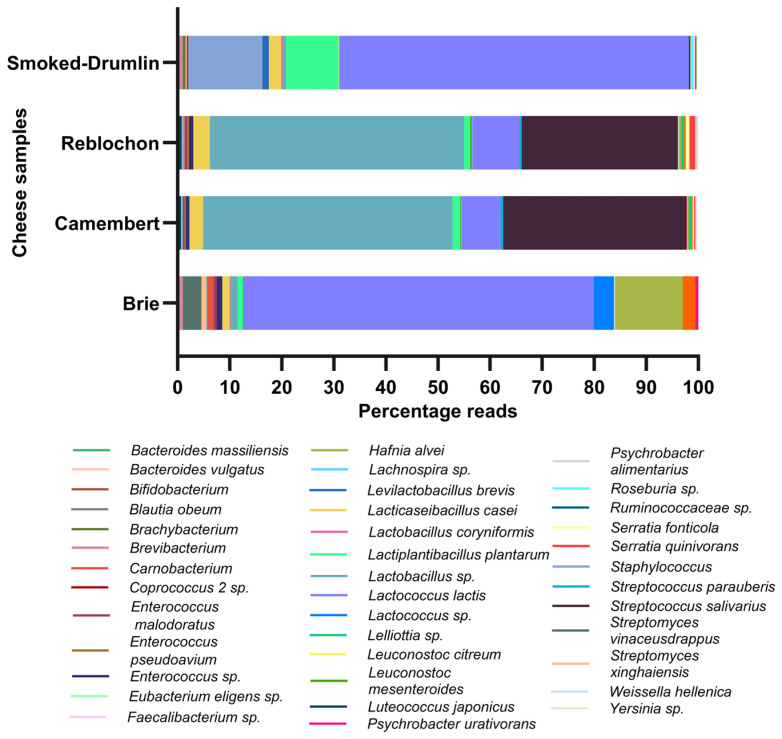
Microbiota composition of four raw milk cheeses. Microbiota composition representation at species level for the four raw milk cheeses (Brie, Camembert, Reblochon, Smoked Drumlin) based on 16S rRNA metagenome sequencing outputs. Each species is color-coded according to the legend below the chart. The chart shows dominant LAB (*Lactococcus*, *S. salivarius* and lactobacilli) and highlights high abundance of *Hafnia* in the Brie cheese sample.

Based on 16S rRNA-based metagenomic analysis, the microbiota of the analyzed Camembert and Reblochon cheeses were dominated by *Streptococcus salivarius* and *Lactobacillus delbrueckii*, while those of the Brie and Smoked Drumlin cheeses were dominated by *L. lactis* (Figure 1). The mesophilic production system applied to Brie and Smoked Drumlin is consistent with the finding of mesophilic lactococci and *Leuconostoc* spp. as major components of their microbiota. Similarly, since Reblochon is typically produced using a mixture of mesophilic and thermophilic production steps, the high abundance of *S. salivarius* (which may likely be represented by *S. thermophilus*) is consistent with the production regime. Conversely, since Camembert is usually produced under mesophilic conditions, the microbiota composition was noteworthy (Figure 1). Furthermore, *Lb. plantarum* and *Lacticaseibacillus casei* were present in low abundance in all four cheeses and may comprise part of the non-starter LAB microbiota. Notably, there appeared to be an abundance of *Hafnia* reads in the Brie sample when compared to those representing the other cheeses, being consistent with the isolation of *Hafnia* strains from this cheese sample (Figure 1 and Supplementary Table S1).

### 3.2. Thermophilic Culture-Based Analysis Increases Selection for Lactic Acid Bacteria

The culture-based analysis of the four raw milk cheeses described above revealed a diversity of non-LAB isolates, particularly on LM17 agar at 30 °C. Therefore, to establish if higher temperatures would yield a more selective LAB composition on media that is designed to enrich for LAB, we analyzed the LAB culturable counts of eight additional raw milk and pasteurized milk cheeses on LM17 agar at 42 °C. Thermophilic counts across all eight cheeses were approximately 10^5^ cfu/mL (Supplementary Table S2). Subsequently, 22 representative colonies were picked and purified from the eight additional cheese samples for speciation using 16S rRNA gene sequencing revealing a more selective enrichment for LAB species including *S. thermophilus*, *L. lactis*, *Enterococcus. faecalis*, *Enterococcus. faecium*, *Pediococcus acidilactici*, and *Lacticaseibacillus paracasei* (19 of 22 isolates were identified as LAB; (Supplementary Figure S2). Interestingly, three isolates were identified as *H. alvei/paralvei* from the second Brie and Camembert samples being consistent with the cultivation-based analysis of the first four raw milk samples. The isolates derived specifically from the pasteurized milk cheeses were all identified as LAB (Table 1 and Supplementary Figure S3).

### 3.3. Hafnia Are Prevalent in Raw Milk Cheeses & Produce Gas from Lactose Metabolism

*Hafnia* strains were isolated exclusively from raw milk cheeses evaluated in this study, i.e., Brie, Camembert and Reblochon cheese samples, i.e., *Hafnia* was absent in pasteurized milk-derived cheeses evaluated in this study. In total, twelve *H. alvei/paralvei* strains were isolated and 16S rRNA gene sequence analysis established that among these were ten *H. paralvei* strains (five of which were isolated from the two Brie samples, three from the two Camembert samples and two from Reblochon and named CO1 through to CO9 and CO12) and two *H. alvei* strains (both isolated from the Reblochon sample and named CO10, CO11). The *Hafnia* isolates were evaluated for their growth capability in different media and were shown to reach higher optical densities in LM17 broth than in LB broth after 24 h incubation at 37 °C (Supplementary Figure S4).

**Figure 2:**
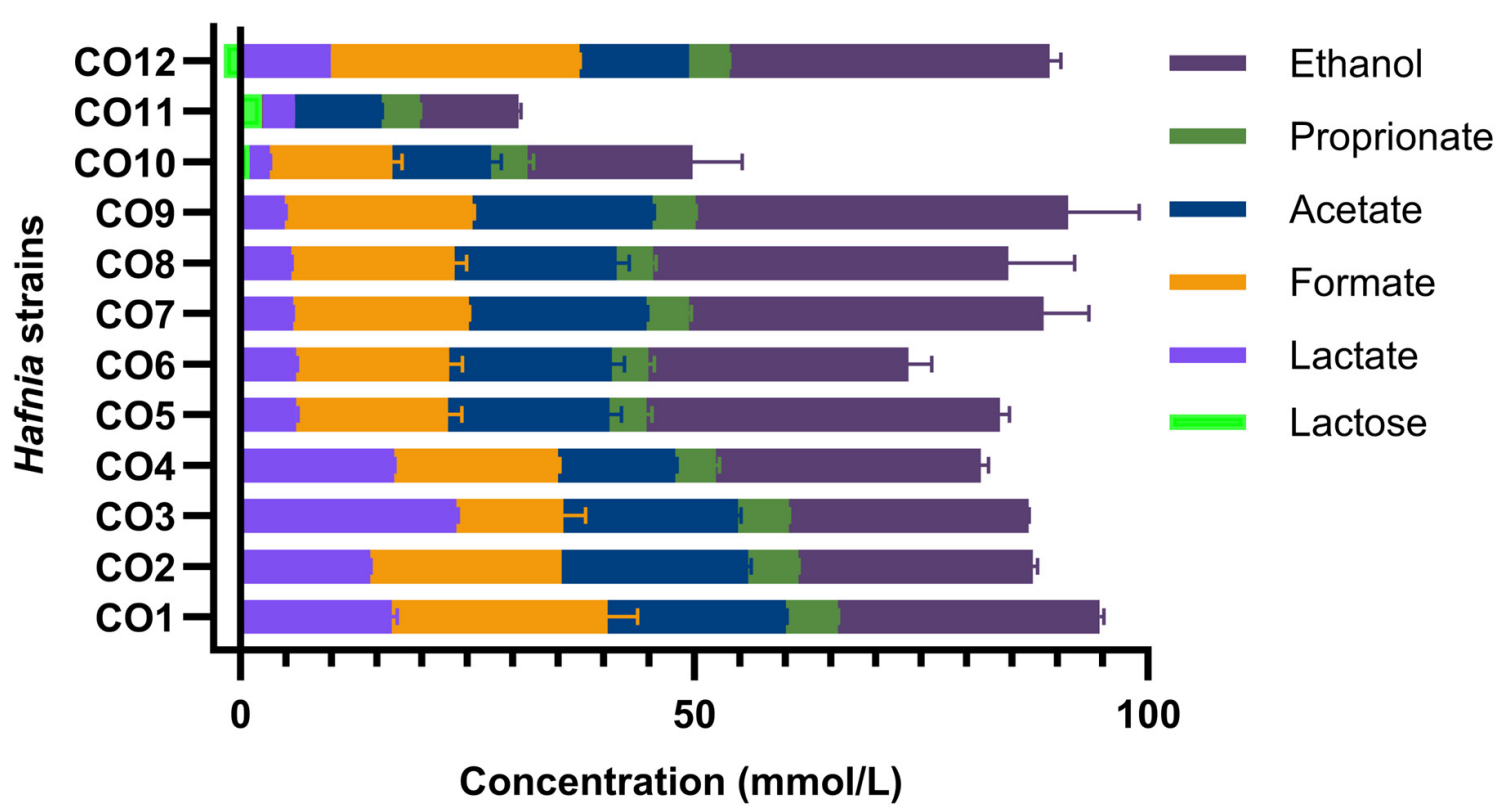
Organic acid production by *Hafnia* isolates. The concentration of organic acids (mmol/L) produced by *Hafnia* spp. isolates grown in LM17 broth (relative to a media control) revealed the production of lactic acid, acetic acid, formic acid as well as a low concentration of propionic acid by all strains apart from CO11 in addition to ethanol. CO11 was not observed to produce formate.

The colony morphologies of all strains at 30 °C and 37 °C on LM17 agar were observed to be large, glossy and creamy white in appearance (Supplementary Figure S5). *Hafnia* strains produce gas in LM17 broth (Supplementary Figure S6) due to the metabolism of the available lactose, while in LB broth, gas production is not observed. Furthermore, the ability to utilize lactose and subsequent conversion into organic acids was evaluated using high-performance liquid chromatography (HPLC) (Figure 2) and phenotypic evaluation on MacConkey agar (Supplementary Figure S7). *H. alvei* strains CO10 and CO11 did not appear to metabolize lactose on MacConkey agar (this being consistent with a lack of gas production in broth assays), whereas the *H. paralvei* isolates produced pink colonies indicating that lactose utilization had occurred (Supplementary Figure S7).

Ethanol was produced by all strains in addition to proprionate, acetate and lactate (Figure 2). Beyond lactose utilization, it was unclear if the *Hafnia* isolates could grow in milk through the metabolism of milk proteins, for example. Therefore, we evaluated the ability of the twelve *Hafnia* strains to grow in milk and their acidification capacity. The initial pH of the milk was 6.5 while the pH of the milk inoculated with *Hafnia* isolates dropped by at least one pH unit after 24 h (Table 3). Two LAB strains isolated in this study (*S. thermophilus* COSt11 and *L. lactis* LL1) were used as positive controls for the assay and were also shown to fully coagulate and acidify the milk within 24 h of the assay as expected.

**Table 3:** Acidification of milk by *Hafnia paralvei* strains relative to lactic acid bacterial control strains after 24 h.

Strain	pH (24 h)
*H. paralvei* CO1	5.3 ± 0.1 ^a^
*H. paralvei* CO2	5.2 ± 0.23 ^a^
*H. paralvei* CO3	5.4 ± 0.17 ^a^
*H. paralvei* CO4	5.4 ± 0.12 ^a^
*H. paralvei* CO5	5.3 ± 0.20 ^a^
*H. paralvei* CO6	5.3 ± 0.15 ^a^
*H. paralvei* CO7	5.4 ± 0.12 ^a^
*H. paralvei* CO8	5.3 ± 0.10 ^a^
*H. paralvei* CO9	5.2 ± 0.23 ^a^
*H. paralvei* CO12	5.2 ± 0.15 ^a^
*S. thermophilus* COSt11	4.0 ± 0.0 ^b^
*L. lactis* L.L1	3.8 ± 0.12 ^b^

Mean pH values of milk inoculated with *H. paralvei* strains (CO1–CO9 and CO12) and control LAB strains (*L. lactis* L.L1 and *S. thermophilus* COSt11) after 24 h of incubation. Values are presented as mean ± standard deviation (n = 3). Different superscript letters indicate statistically significant differences between strains as determined by one-way ANOVA followed by Tukey’s post hoc test (*p* < 0.05).

### 3.4. Hafnia Strains Are Tolerant to a Wide Range of Growth Conditions

**Figure 3:**
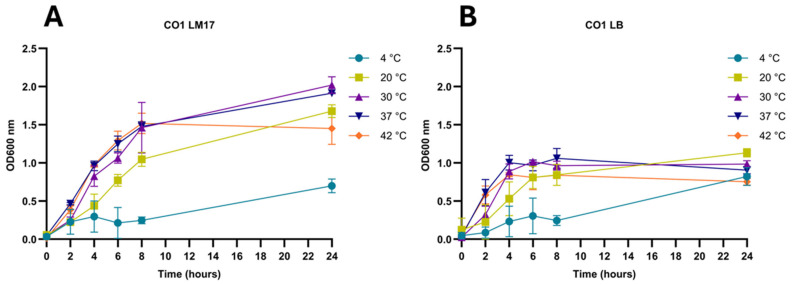
Temperature-dependent growth of *H. paralvei* CO1. Growth profiles optical density (OD600) of representative strain *H. paralvei* CO1 at different temperatures (4, 20, 30, 37 and 42 °C) in both LM17 (**A**) and LB (**B**) broth. Notably, higher optical densities are achieved in LM17 broth than in LB broth, which is observed for all evaluated *Hafnia* strains (Supplementary Figure S4).

**Figure 4:**
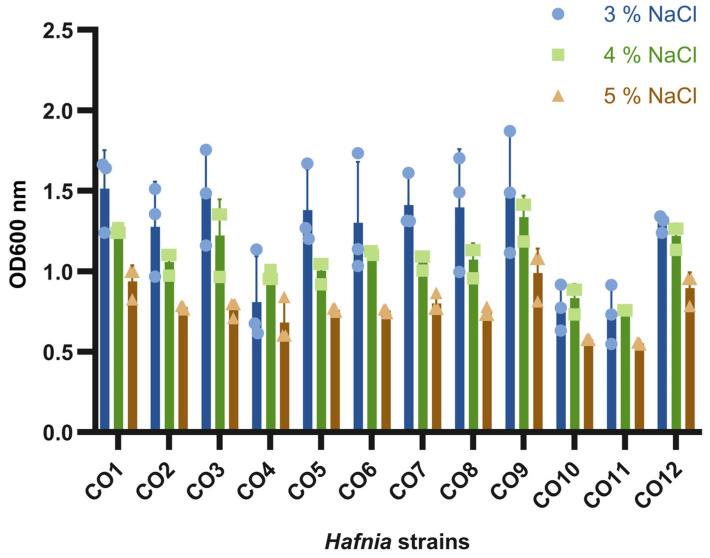
**Salt tolerance of *Hafnia* strains.** Graph depicting the optical density (OD600) after 24 h incubation of the twelve *H. paralvei* and *H. alvei* strains in LB broth supplemented with 3–5% salt. All strains were observed to tolerate all salt concentrations although with reduced optical density (OD600) with increasing salt concentrations.

All *Hafnia* strains were capable of growth across the range of temperatures evaluated in this study (4–42 °C) in both LM17 and LB broth (Figure 3 and Supplementary Figure S4). *H. alvei* strains, however, grew relatively slower than the ten *H. paralvei* strains while they are also capable of growth in both LB and LM17 broth. Notably, the strains were observed to achieve higher optical densities in LM17 than in LB broth, which is likely due to exopolysaccharide production and/or biofilm formation in LM17 broth. This higher optical density in LM17 broth coincided with the presence of a thick pellicle on the surface of the LM17 broth that was not observed in LB broth and which may represent the liberated exopolysaccharides as part of extracellular matrices, which have previously been reported in other Gram-negative species [37]. It is noteworthy that the extent and timing of observed pellicle formation is strain-dependent. *Hafnia* strains were also obsereved to tolerat salt conditions of 3–5 % added to LB broth (Figure 4).

### 3.5. Hafnia Strains Do Not Exhibit Antagonistic Interactions with Lactic Acid Bacteria

*Hafnia* strains are known to be motile. In the present study, the *Hafnia* isolates displayed different extents of motility on LB and LM17 agar, respectively (Supplementary Figure S8A–D). Greater motility was observed on LM17 relative to LB. Eiken agar motility assays were also conducted on all 12 *H. paralvei/alvei* strains to define the type of motility. Three *H. paralvei* strains displayed swarming motility type (Supplementary Figure S8C), while seven *H. paralvei* and *H. alvei* strains displayed swimming motility type (Supplementary Figure S8D), and two *H. paralvei* strains displayed both swarming and swimming motility capabilities, respectively.

The ability of the *Hafnia* isolates to grow on LM17 agar and their motility phenotype on this medium prompted us to evaluate possible interaction (antagonistic or symbiotic) with LAB derived from the raw milk cheeses. To explore this phenomenon, we co-inoculated (by spot plating) the individual *Hafnia* isolates with two individual LAB isolates emanating from this study, i.e., a *S. thermophilus* and a *L. lactis* isolate. Using *H. paralvei* CO12 as a representative, it was evident that the LAB isolates did not antagonize the *Hafnia* isolate, as the motile direction was such that the *S. thermophilus* and *L. lactis* spot cultures were engulfed (Supplementary Figure S9A–F). Therefore, it appears that there is possible cooperation or at the least no obvious antagonism between the evaluated LAB and *Hafnia* isolates.

## 4. Discussion

The microbiota of a range of PDO and non-PDO of Italian raw milk-derived cheeses has been analyzed using metagenomics approaches, including Pecorino, Caciocavallo and Mozzarella [9,21]. From these studies the dominant identified species include *S. thermophilus*, *L. lactis*, *Lb. plantarum* and *L. mesenteroides* [21,38]. Culture-dependent approaches have been applied in other studies to evaluate raw milk and pasteurized cheese isolates, e.g., gouda, grana-like cheese, soft cheeses from bovine and ovine raw milk, identifying LAB such as *Lactococcus* spp. and *Leuconostoc* spp. as the most dominant species [39,40,41]. Since each approach has benefits and limitations, applying both methodologies presents an optimal scenario for defining the microbial communities and the functional importance or relevance of the component members. Here, we have taken a combined approach to view the microbial complexity of four raw milk-derived cheeses and established that in addition to the core LAB component, multiple other species were present that may contribute to the organolepsis of the individual cheeses.

As the numbers of artisanal raw milk cheese producers appear to be increasing, in-depth understanding of microbiota present in raw milk used to produce cheeses is needed. Only a small number of studies have applied the combination of culture-dependent and independent approaches to study these complex bacteria environments [42,43,44]. For example, raw milk is known to harbor *Enterobacteriaceae* and a recent study of four Polish Twarog cheese samples identified *E. coli* and *H. alvei* in the metagenomes of two of the four samples in addition to LAB species [45]. Furthermore, the culture-based analysis permitted insights into the viability of the coliform/*Hafnia* counts to understand the implications of their presence on the quality, safety and consumer acceptability of the final product. Similarly, we suggest that partnering metagenomics with culture-dependent analysis in identifying species in raw milk is beneficial to define the true complexity of these products and to understand (a) acceptable threshold limits of the presence of organisms such as *Hafnia* spp. and (b) how we may apply more deliberately strains of non-LAB species for their functional properties in the future in precision fermentation approaches.

The functional role of LAB and their contributions to dairy fermentations are well studied [46,47]. In the context of raw milk-derived cheeses, the diversity and contributions of organisms beyond the LAB are less well interrogated [9,21,22,48]. *H. alvei* has recently been linked to cheese ripening and likely beneficial interaction with *Debaromyces hansenii* and *Brevibacterium aurantiacum* with the three organisms described as a “ripening culture” in smear-ripened cheeses [49]. *H. alvei* and *B. aurantiacum* were positively associated with the production of volatile sulfur compounds being desirable for use in commercial cultures for aroma development in cheeses [49]. *H. alvei* was also observed to vigorously stimulate growth of *B. aurantiacum* by eight to 10-fold within 28 days of cheese manufacturing and was also shown to provide iron to *B. aurantiacum* (possibly by siderophore production) [49]. Siderophore systems enable competitive or cooperative nutrient sharing among microorganisms. Since iron is poorly soluble, Gram-negative bacteria produce siderophores molecules which bind to iron to manage iron depletion or overload to prevent cell damage [50]. *Hafnia* spp., like other enteric bacteria [51], likely form siderophore–iron complexes that chelate iron from their surroundings and these siderophores can also be transported back into the cell via ABC transport systems and TonB-dependent outer membrane receptors [52]. The mechanism used by bacteria for cross-feeding is xenosiderophore utilization where one bacterium will use siderophores produced by a different species if they have compatible receptors to import the same iron–siderophore complexes [53]. *H. alvei* spp. are reported to encode a hydroxamate-type siderophore cluster which is distinct from classical cate-cholate or carboxylate systems [49,54]. Bacteria capable of encoding exogenous siderophore are reported to have a competitive advantage of siphoning multiple iron chelating molecules in the environment [55]. Expression of this cluster is reported to stimulate iron availability in cheese minicultures, influencing iron availability in mixed cultures which explains the reported beneficial interaction between *H. alvei* and *B. aurantiacum* through provision of iron [49]. *B. aurantiacum* is reported to stimulate the growth of *H. alvei* through production of proteases and lipases which generate energy substrates for *H. alvei* [49]. However, further investigation is needed to validate the interaction of these two species in cheese ripening. Conversely, strain *H. alvei* H4 (a wild-type species) has been associated with spoilage of chilled aquatic foods [56,57]. It is mainly isolated from spoiled foods such as fish, raw milk, chicken, ground beef with its spoilage properties being linked to quorum sensing (QS) through the production of *N*-(3-oxohexanoyl)homoserine lactone, *N*-butyryl-homoserine lactone and *N*-hexanoyl-dl-homoserine lactone (types of acyl-homoserine lactone signaling molecules) that stimulate biofilm formation [58]. *H. alvei/paralvei* species have Luxl/LuxR-type quorum sensing systems that produce the signaling molecules N-Acyl homoserine lactones (AHLs) [59]. Luxl-type synthase produces AHLs that accumulate when the bacteria population reaches a high density. These molecules bind to the LuxR-type transcriptional regulator which will activate specific gene pathways including biofilm formation, virulence-associated gene expression and motility, resulting in biofilm formation on cheese surfaces or in curd matrices [60,61]. QS has been linked to biofilm formation in *Hafnia* although precise gene networks involved are yet to be fully characterized [62]. Biofilm formation also occurs when QS molecules stimulate synthesis of extracellular polysaccharide structures (*EPS*). In the current study, pellicle formation (which is associated with *EPS* production) was observed in the *Hafnia* isolates when growing in rich media such as LM17. *EPS* production further stabilizes biofilm structures, enabling bacteria to retain nutrients and protecting cells from external stresses including changing environmental conditions such as those experienced during cheese production and ripening. QS also facilitates motility gene expression in *Hafnia* bacteria [60,62]. When cell numbers are low, flagella-associated gene expression increases leading to swimming or swarming motility phenotypes while at high cell density, motility is reduced and biofilm formation is favored [62]. QS also facilitates interactions between microbes to aid cooperation in cheese ripening and it also enables microbes to colonize curd pores which would be beneficial to production of high quality cheese [49,63].

QS in *H. alvei* has also been linked to the regulation of proteolytic pathways as well as the production of both acidic and alkaline metabolites in this species [57], thus enabling it to better survive in stressful conditions [57]. In the current study, we present a detailed analysis of the microbiota in raw and pasteurized milk cheeses (Table 1) using both culture-dependent and independent approaches. We characterized *Hafnia* isolates and their seemingly non-antagonistic relationship with LAB (*L. lactis* and *S. thermophilus*) and the precise nature and extent of co-operation between strains of these organisms will be the subject of ongoing investigation (and is therefore beyond the scope of this manuscript).

Two strains of *H. alvei* isolated from Spanish raw ewe’s milk PDO cheeses have recently been proposed as adjuncts due to their proteolytic activity at low temperature during ripening and low gas production characteristics [14]. However, the direct and deliberate application of *Hafnia* spp. in food fermentations is nuanced as they have been isolated from different environments including soil, food, human and animal feces, and water [64]. They have been described as commensal organisms [65], while they have also been reported to exhibit opportunistic pathogenic potential in some cases [59,66,67,68,69]. Several studies [66,70,71,72,73,74] have identified infections in humans that have been associated with *H. alvei*, including urinary tract infections (pyelonephritis); sinus tract infection in open fractures; hospital acquired pneumonia; possible osteomyelitis; reactive arthritis; cholangitis; cholecystitis; appendicitis; septicemia and three deaths reported as having been caused by *H. alvei* infections [66]. Patients were mostly those receiving care in hospitals with underlying illnesses including diabetes, malignancy or recently undergone surgery [70,73]. *H. alvei* species have also been associated with cheese spoilage at ripening stages [56,,15,75]. *H. alvei* species have been reported to have the ability to decarboxylate lysine and ornithine in cheese [56], which is associated with the production of unpleasant odors and undesirable flavors. Therefore, while there are many possible benefits from introducing strains of this species in foods, we speculate that much more information will be needed regarding threshold values, storage conditions and the specific products to which they may be added.

Two other studies have reported the isolation of *Hafnia psychrotolerans* from marine environments and fish products where it is deemed a foodborne pathogen [76,77]. Conversely, *H. paralvei* has not been well characterized in terms of its contribution to aroma, texture, ripening or improving quality of cheese and other dairy products nor its impact on food deterioration/spoilage. *H. paralvei* strains were shown to be the most frequently isolated bacteria in all cheese samples, with a total of ten *H. paralvei* isolates compared to two *H. alvei* isolates in this study. Since *H. paralvei* species have been less studied than *H. alvei* species, it is important to understand how they behave in food matrices and most importantly how they interact with other microbes, especially LAB. Based on evidence in the present study, *H. paralvei* strains have a broader temperature and salt tolerance than *H. alvei* species with possible implications for their adaptability in the harsh conditions associated with ripening.

In high salt conditions such as those associated with cheese production, stress responses are triggered and impact the expression of genes including *proP* and *proU* which encode transport systems for compatible solutes, *otsA/otsB*, involved in trehalose biosynthesis, associated with motility acid production and metabolic activities that help in survival of the bacteria cell [78,79,80]. *Hafnia* isolates in this study were observed to be resistant to up to 5% NaCl allowing them to persist. Enteric bacteria in high NaCl concentrations respond through conserved osmotic stress response systems such as EnvZ/OmpR [79]; however, specific stress response pathways have not been extensively characterized in *Hafnia*, highlighting the need for further investigation of such phenomena in this genus. Another enteric bacterial response is to induce compatible solute transport systems to also adjust transcription for cell survival and stability [78,79,81,82]. Motility is suppressed to maintain energy in the cell when salt levels are elevated [80]. This is achieved through the downregulation of genes associated with flagellar biosynthesis. As mentioned earlier, studies in *Enterobacteriaceae* have shown that osmotic stress can lead to downregulation of motility genes which may facilitate biofilm formation and surface attachment; however, relevant studies demonstrating this mechanism in *Hafnia* are limited [80]. Salt stress may alter relative flux through central metabolism, potentially changing acid production profiles rather than initiating acid production itself [80,81,82]. Mixed acid fermentation is likely to be differentially regulated during the physiological adaptation to osmotic stress. Cells will redirect metabolic flux to energy efficient fermentation pathways and if redox balance is also affected, production of organic acids including lactate and acetate is also altered which will ensure pH stability for cell survivability [79]. *Hafnia* cells may counter osmotic pressure by accumulating compatible solutes like proline and trehalose which indirectly modify acid production [82]. These solutes are commonly accumulated by *Enterobacteriaceae* during osmotic stress; however, their specific role in *Hafnia* within cheese systems has not been directly shown. It would be beneficial to fully understand the mechanisms of each *Hafnia* species in cheese productions. Furthermore, based on data from this and previous studies, the apparent consistent presence of *Hafnia* strains in raw milk-derived cheeses and the long history of (typically) safe consumption of these products, it seems likely that *Hafnia* strains are unlikely to pose a significant threat to a healthy consumer [49]. However, additional and systematic research is required to evaluate the risk to vulnerable cohorts in society.

If lactose had not been fully exploited by LAB in the initial fermentation, we propose that *H. paralvei* may have the opportunity to “bloom” in the storage and ripening phases, although it should also be considered that they may produce gas that may be associated with product defects, particularly in hard or semi-hard cheeses. Recently, *H. alvei* was identified as a dominant component of the spoilage microbiota (72.1%) of a cheese derived from raw goat’s milk where early blowing was observed [15]. Conversely, gas production (carbon dioxide) could have bio-preservative effects by reducing the oxidation-reduction potential of the product that would impact the growth of aerobic spoilage and pathogenic bacteria. Furthermore, *H. paralvei* isolates were observed to acidify milk (Table 3), reducing the pH, thereby contributing to the exclusion of spoilage/pathogenic organisms. In general, *Hafnia* are described as lactose-negative although there are limited reports of strains that possess lactose utilization-associated genes on their plasmids [83]. In *Enterobacteriaceae*, these genes are mostly encoded on the chromosome, and *Hafnia* spp. genetic analyses indicate the presence of LacZ and LacY homologs in several sequenced strains, indicating that lactose metabolism occurs via a similar system [84,85,86]. However, this trait is not uniformly distributed across all *Hafnia* strains and the genomic organization and regulatory control of the *lac* operon in *H. paralvei* remain poorly characterized and the comparative differences between *H. paralvei* and *H. alvei* are limited. Lactose metabolism in these strains is supported by phenotypic evidence and limited genomic data analyses. Some *H. alvei* spp. on the other hand, have been reported to express variable lactose fermentation phenotypes [87]. These may arise from differences in gene presence, expression levels or regulatory control; however, this requires further investigation.

Furthermore, *Hafnia* spp. facilitate gas production and additional fermentation products including acetate, lactate, ethanol through mixed-acid fermentation during carbohydrate metabolism [88]. Carbohydrates including glucose or galactose from lactose are converted into pyruvate which is then cleaved to acetyl-CoA and formate by pyruvate formate-lyase (PfIB) [89]. The formate can then be converted by a formate hydrogen lyase complex (FHL) to produce CO_2_ and H_2_.

Direct studies of interactions and metabolic cross-feeding between LAB and *Hafnia* during lactose depletion are limited; however, as discussed earlier, there are mini-cheese model experiments using *H. alvei* and other surface microbes that have demonstrate expression of metabolic pathways including D-galactonate catabolism and iron acquisition that suggest collaborated nutrient utilization in a cheese ripening context [49]. Other model cheese systems show that LAB act first in rapid acidification of milk, lower the pH and create an environment for species such as *Hafnia* to colonize after lactose depletion and the LAB also provide metabolites including lactate, peptides used by other non-starter bacteria in the later stages of cheese ripening [90,91]. While many of these studies do not exclusively focus on *Hafnia* and LAB cross-feeding, they demonstrate potential cooperative metabolic interactions relevant to cheese ecosystems, which will likely form the basis of future investigations in this field. Future investigations will seek to understand the possible mutualism that exists between *Hafnia* and LAB in the dairy niche and to define the specific role of *Hafnia* in cheese ripening and/or spoilage. This study sets the foundations to explore and exploit the broader microbiota of raw milk cheeses and to consider the role of non-LAB organisms in this context. We envision that this will culminate in the development of microbiologically based quality markers for artisanal cheese production systems while simultaneously increasing the portfolio of ripening cultures that may be applied in defined starter culture systems in the future.

## 5. Conclusions

Raw milk-derived cheeses have established the dominant presence of LAB including lactococci or *S. salivarius* and with a secondary population of various lactobacilli. However, *Hafnia* are consistently detected as part of the non-starter microbiota in raw milk cheeses, where they may play a role in early and late ripening stages. The evidence presented in this chapter suggests that *Hafnia* spp. exploit alternative substrates and engage in metabolic exchanges to be able to ferment lactose, produce gas, use quorum sensing molecules for communication, cooperation and/or to compete for nutrients, which might influence their succession as compared to other microorganisms, their ability to form biofilms and overall metabolic activities during cheese production and ripening. Genomic analyses suggest that *H. paralvei* have homologs of lac operon genes, though their regulation and expression under cheese ripening conditions remain poorly characterized, and the studies of comparative data for *H. alvei* are limited. Consistent with mixed-acid fermentation pathways common to *Enterobacteriaceae*, *Hafnia* produce gas and organic acids, although the contribution to acidification is minor relative to LAB such as *S. thermophilus* and *L. lactis*. Regulatory networks known in enteric bacteria, including CRP–cAMP, FNR, and ArcA/ArcB, likely influence metabolic activity and gas production, but their roles in *Hafnia* remain largely unexplored. Osmotic stress responses, including compatible solute accumulation, and quorum sensing via LuxR-type regulators may modulate motility and biofilm formation, yet direct evidence in cheese matrices is currently lacking. Importantly, *Hafnia* may engage in metabolic interactions with LAB playing a role in the complexity of microbial communities during cheese ripening potentially through mechanisms including cross-feeding of lactose-derived or secondary metabolites, stress adaptation, interspecies signaling, nutrient cycling and microbial community dynamics without directly driving acidification. Future studies comparing *H. paralvei* and *H. alvei* will need to clarify species-specific metabolic capabilities, regulatory mechanisms, and interactions with LAB, providing a more comprehensive understanding of *Hafnia* spp. in dairy food ecosystems.

## Figures and Tables

**Table 1:** Details of the 12 cheeses selected for the study including their animal origin, texture and maturity level.

Product	Origin of Animal Milk	Texture	Milk State	Time Post-Production
Fleur du Maquis	Sheep	Soft	Pasteurized	3–6 weeks
Brie (n = 2)	Cow	Soft	Raw milk	1 week–3 months
Saint Felicien	Cow	Soft	Pasteurized	9 days
Mozzarella	Buffalo	Semi-soft	Pasteurized	0 days (fresh)
Caciocavallo	Cow	Hard	Pasteurized	3–6 months
Camembert (n = 2)	Cow	Soft	Raw milk	1 week–3 months
Pecorino (n = 2)	Sheep	Semi-hard & hard	Pasteurized	1–6 months
Smoked Drumlin	Cow	Hard	Raw milk	1–3 weeks
Reblochon	Cow	Semi-soft	Raw milk	1–3 weeks

**Table 2:** Viable counts of the four raw-milk cheeses selected for metagenomic and culture-dependent analysis.

Medium	TSA (cfu/mL)		LM17 (cfu/mL)		MRS (cfu/mL)		MacConkey (cfu/mL)
Temperature	30 °C	37 °C	30 °C	37 °C	30 °C	37 °C	37 °C
Brie	2.2 × 10^7^	2 × 10^7^	2.32 × 10^7^	2.07 × 10^7^	1.45 × 10^6^	6.5 × 10^6^	7.3 × 10^6^
Camembert	2.3 × 10^6^	3.9 × 10^6^	5.9 × 10^6^	4.9 × 10^6^	6.1 × 10^6^	5.8 × 10^6^	4 × 10^5^
Smoked Drumlin	5.4 × 10^6^	4.4 × 10^6^	2.95 × 10^7^	1.53 × 10^7^	4.5 × 10^6^	2.8 × 10^6^	0
Reblochon	3.7 × 10^7^	1.4 × 10^7^	3.13 × 10^7^	2.9 × 10^7^	1.73 × 10^7^	1.14 × 10^7^	8.4 × 10^5^

## Data Availability

The metagenomics data of four raw milk cheeses; Brie, Camembert, Reblochon and Smoked Drumlin have been deposited in the GenBank database. The raw sequences of the 16S rRNA microbial profiling of each of the four cheese samples are available through the Bioproject accession number PRJNA1345581.

## Supplementary Materials

The following supporting information can be downloaded at: https://www.mdpi.com/article/10.3390/foods15071160/s1. Supplementary Table S1: Culture-dependent isolates of the four raw milk cheeses in comparison to culture-independent analysis; Supplementary Table S2: Viable counts on LM17 agar from the eight additional cheeses for LAB species analysis; Supplementary Figure S1: Microscopic evaluation of *Hafnia* cells, A-strain CO3 grown on LM17 agar, B-strain CO1 grown on LB agar; Supplementary Figure S2: Species identification of the 22 isolates from eight cheeses based on 16S rRNA gene sequencing; Supplementary Figure S3: Comparison based on culture-dependent and culture-independent analysis of the 12 analyzed cheeses; Supplementary Figure S4: *Hafnia* temperature growth profiles; Supplementary Figure S5: *Hafnia* colony morphologies; Supplementary Figure S6: *Hafnia* gas production in LM17 broth after 24 h of incubation; Supplementary Figure S7: *Hafnia* lactose metabolism; Supplementary Figure S8: *Hafnia* strains motility; Supplementary Figure S9: *Hafnia* and LAB interaction assays.
