# Supplementary material for: Raw Milk Cheese Microbiomes: A Paradigm for Interactions of Lactic Acid Bacteria in Food Ecosystems
Source: Foods. 2026 Mar 30;15(7):1160. doi: 10.3390/foods15071160 (PMC13073097; doi:10.3390/foods15071160)
Supplement: Supplementary file 1 [file foods-15-01160-s001.zip › foods-4195608-supplementary.pdf]

Supplementary data

Table S1 shows culture-dependent isolates of the four raw milk cheeses in comparison to culture-independent analysis (Fig.1)

Cheeses	Isolate species based on culture-dependent 16S rRNA sequencing
Brie 2	*Enterococcus faecalis* (1)
	Hafnia paralvei (3)
	Lactococcus lactis (3)
Camembert 2	*Levilactobacillus brevis* (1)
	Lactococcus lactis (2)
	Hafnia paralvei (2)
	Enterobacteriaceae bacterium (1)
Reblochon	*Lactococcus lactis* (1)
	Leuconstoc mesenteroides (2)
	Hafnia alvei (2)
	Hafnia paralvei (2)
Smoked Drumlin	*Lactiplantibacillus plantarum* (1)
	Staphylococcus casei (1)
	Staphylococcus equorum (2)

Table S1: Bacterial isolates from the four raw milk cheeses.

Bacterial isolates from the four raw milk cheeses based on culture-dependent analysis show dominance by LAB species, followed by *Hafnia* species.

Table S2: Viable counts on LM17 agar from the eight additional cheeses for LAB species analysis

Cheese	42 °C (cfu/ml)
Fleur du Maquis	7.9×10^5
Brie	2.90×10^5
Saint Felicien	2.87×10^5
Mozzarella	1.3×10^5
Caciocavallo	2.72×10^5
Camembert	3.00×10^5
Pecorino 1	2.98×10^5
Pecorino 2	1.46×10^5

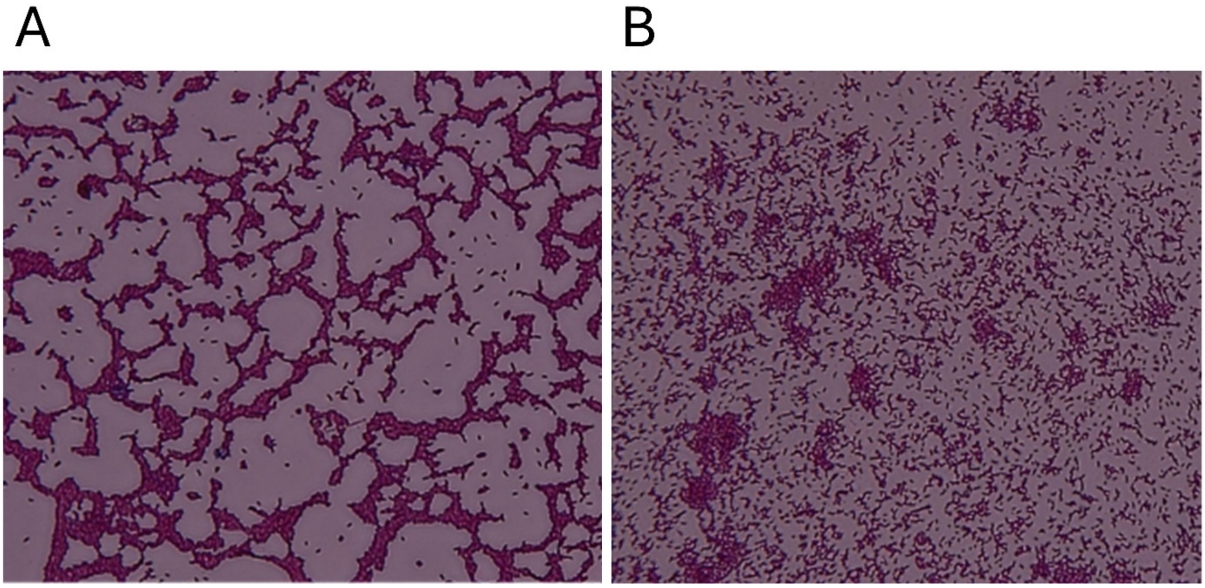

Figure S1: *Hafnia* isolates microscopic analysis.
Microscopic evaluation of representative *Hafnia* isolates CO1 and CO3 grown on LM17 and LB agar showing gram-negative pink rods morphology and aggregation.

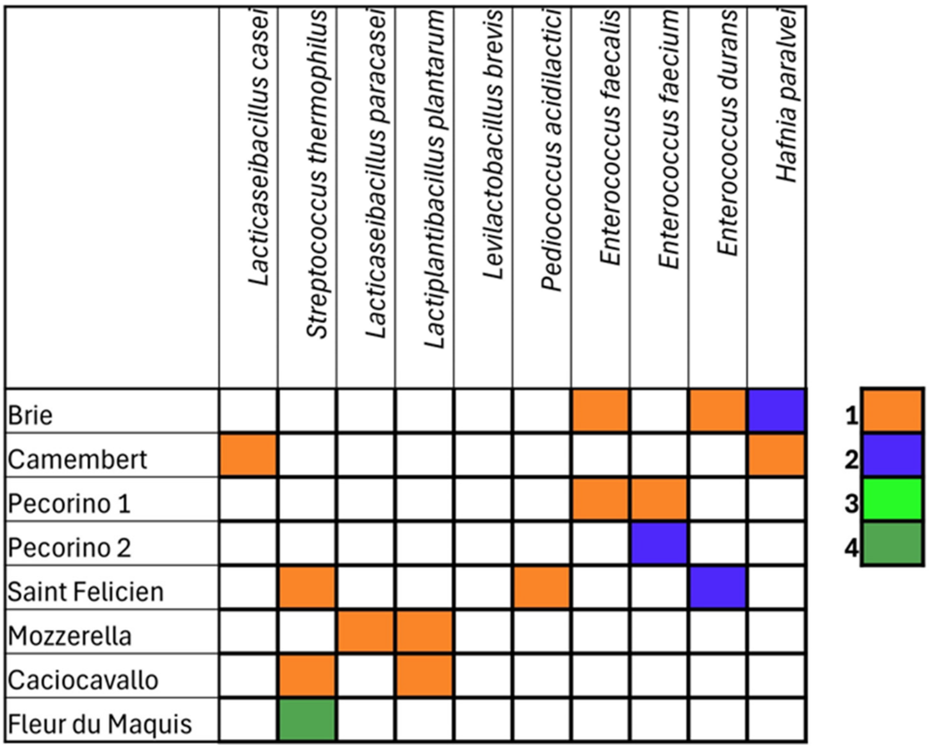

Figure S2: Species isolated from the additional eight cheeses.
Species identification of 22 isolates from eight cheeses based on 16S rRNA sequencing, highlighting selective enrichment for LAB at 42 °C. The number of isolates per cheese is colour-coded as per the legend.

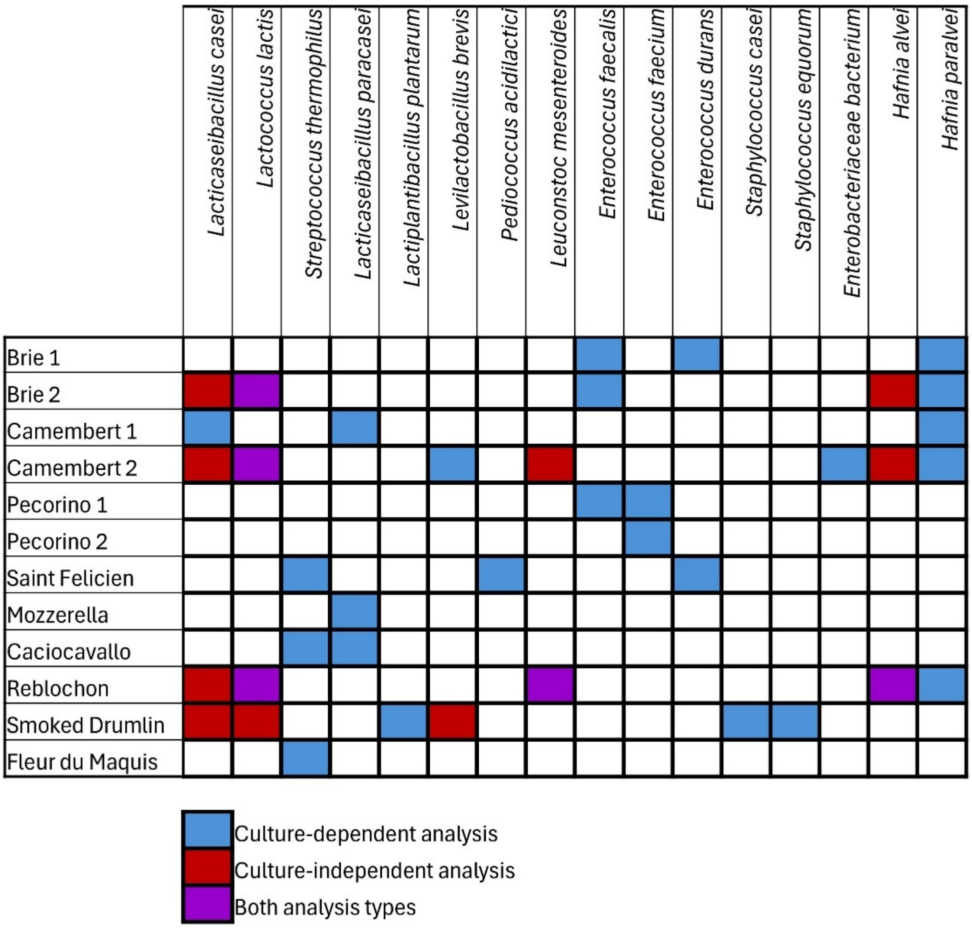

Figure S3: Culture-independent and culture-dependent species comparisons of the 12 analysed cheeses highlighting the species identified using the different approaches or using both approaches as indicated by the colour-coded legend.

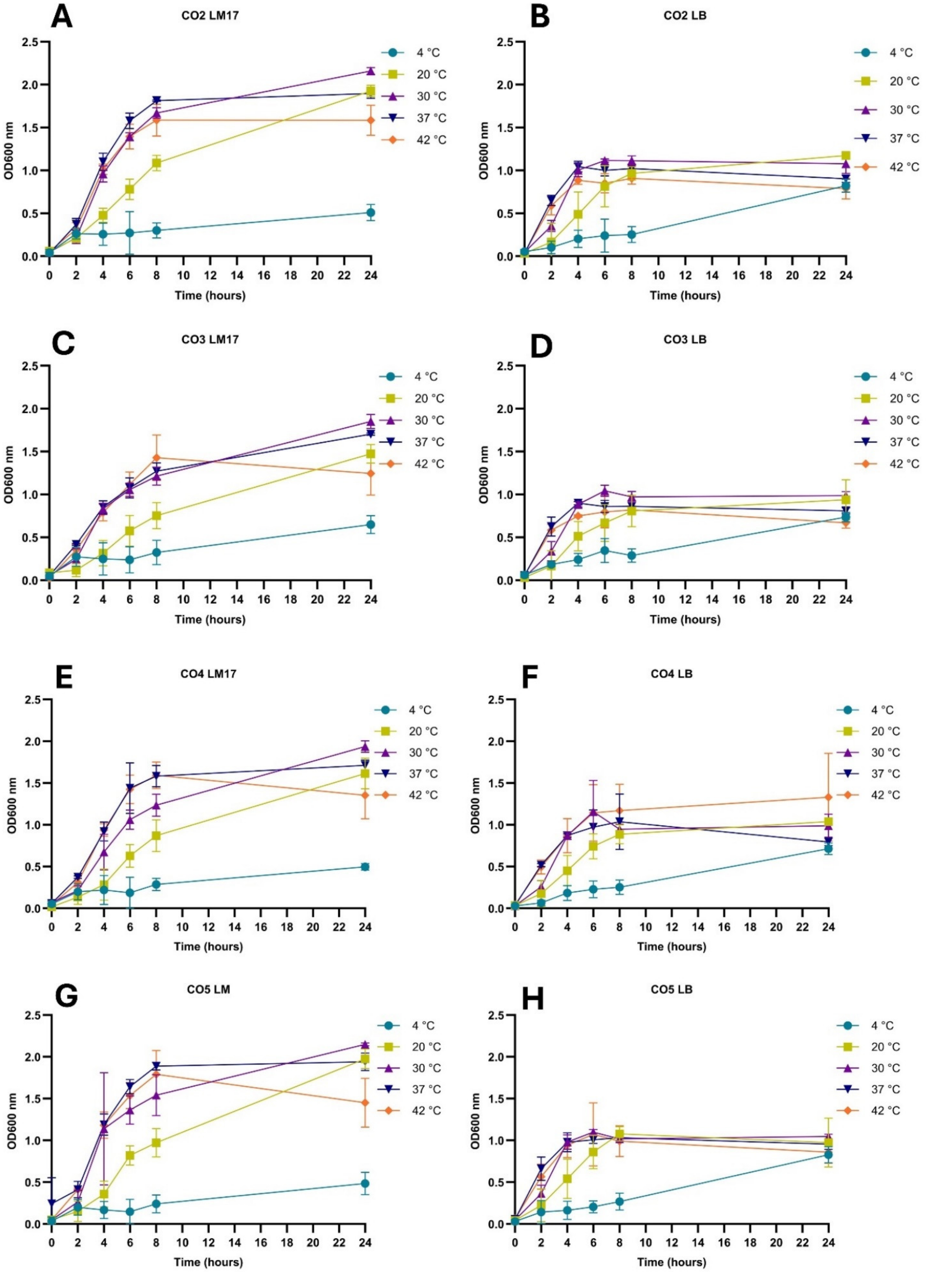
A
CO2 LM17
B
CO2 LB
C
CO3 LM17
D
CO3 LB
E
CO4 LM17
F
CO4 LB
G
CO5 LM
H
CO5 LB
4 °C
20 °C
30 °C
37 °C
42 °C
OD600 nm
Time (hours)

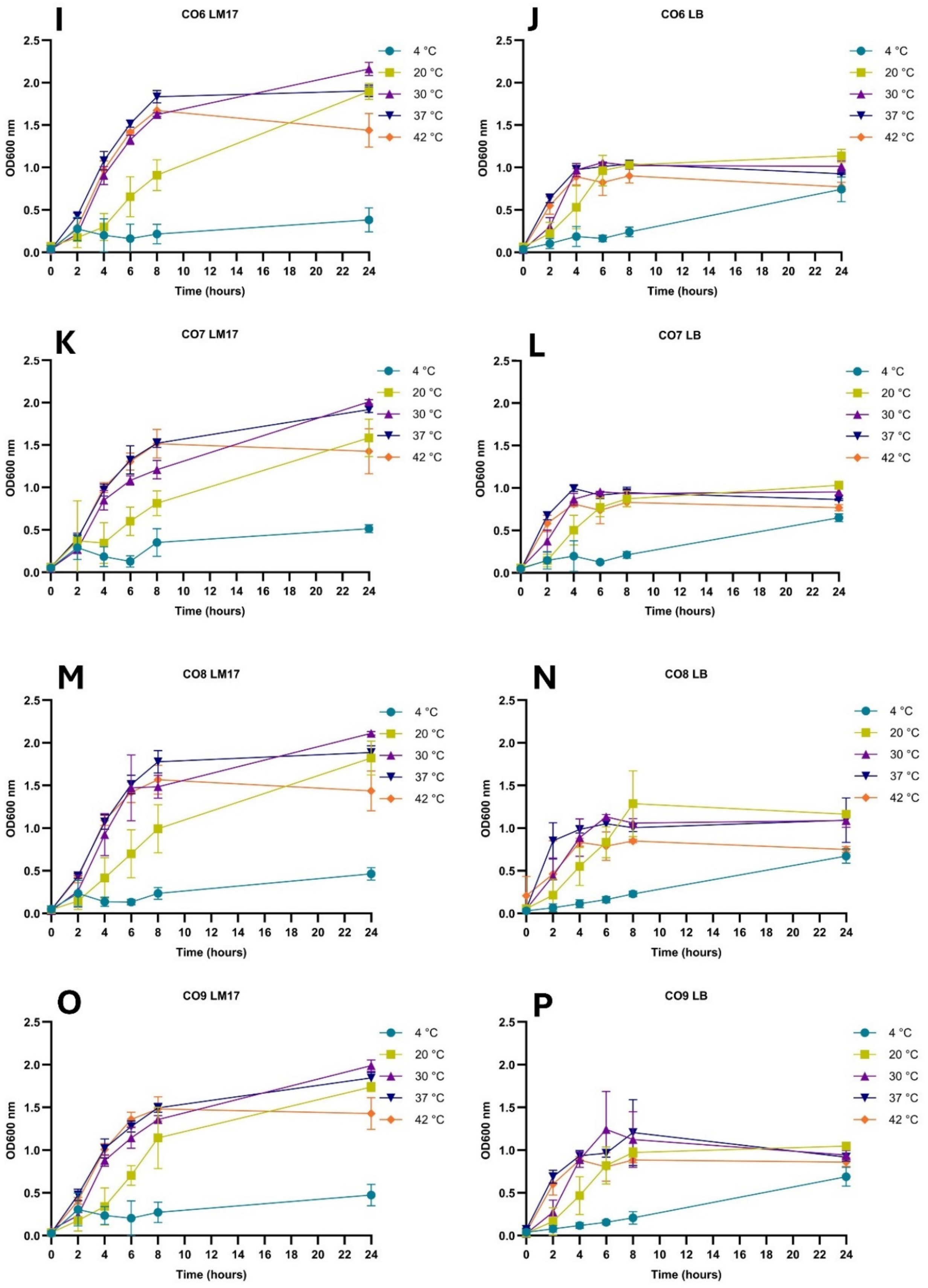

I
CO6 LM17
J
CO6 LB
K
CO7 LM17
L
CO7 LB
M
CO8 LM17
N
CO8 LB
O
CO9 LM17
P
CO9 LB
OD600 nm
Time (hours)
4 °C
20 °C
30 °C
37 °C
42 °C

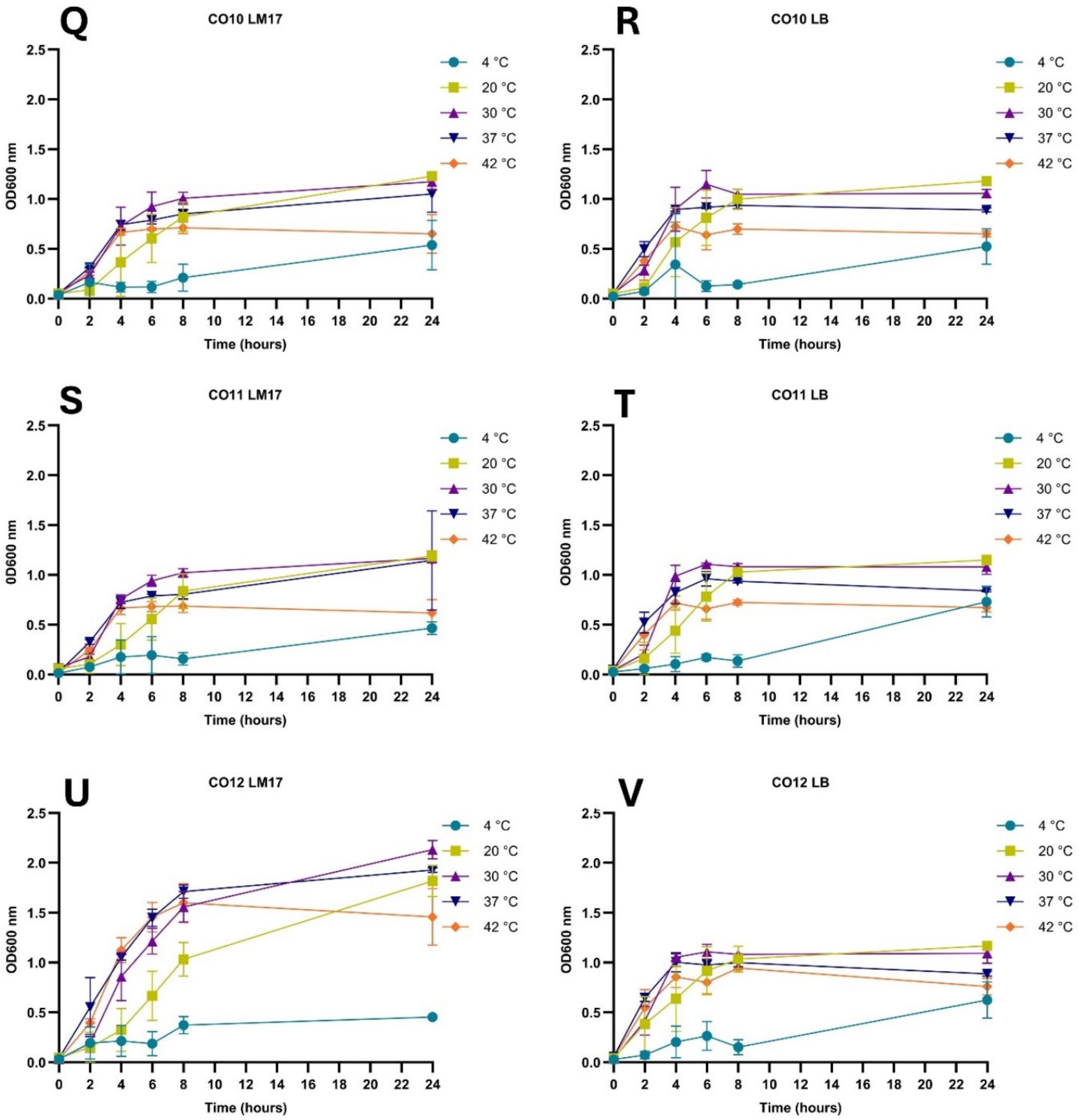

Figure S4: *Hafnia* growth profiles at different temperatures.

Growth profiles of *Hafnia* isolates at multiple temperatures (4 – 42 °C) in LM17 and LB broth show higher optical density (OD600) in LM17 broth than in LB broth for each *H. paralvei* strain (CO1 – CO9 & CO12) apart from *H. alvei* strains (CO10 & CO11) that show relatively similar growth profiles in both media.

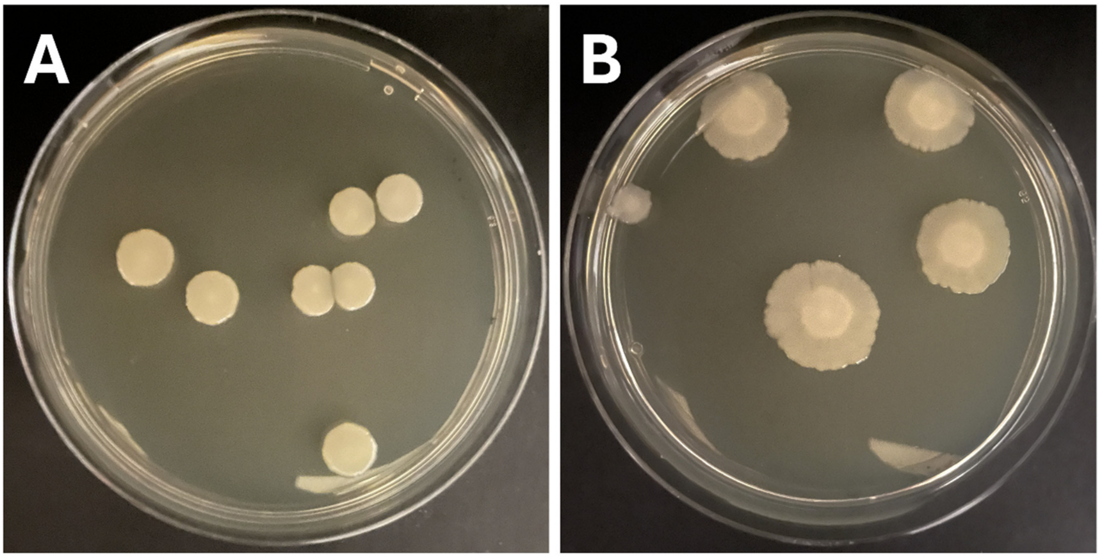

Figure S5: *Hafnia* colony morphology.
Colony morphology of *Hafnia* strains on LM17 agar at 37 °C, showing large, glossy, creamy-white colonies of representative species *H. paralvei* CO1 (A) and *H. paralvei* CO3 (B). *H. paralvei* CO3 unlike other *H. paralvei* strains produces thin transparent extracellular layers around its colonies.

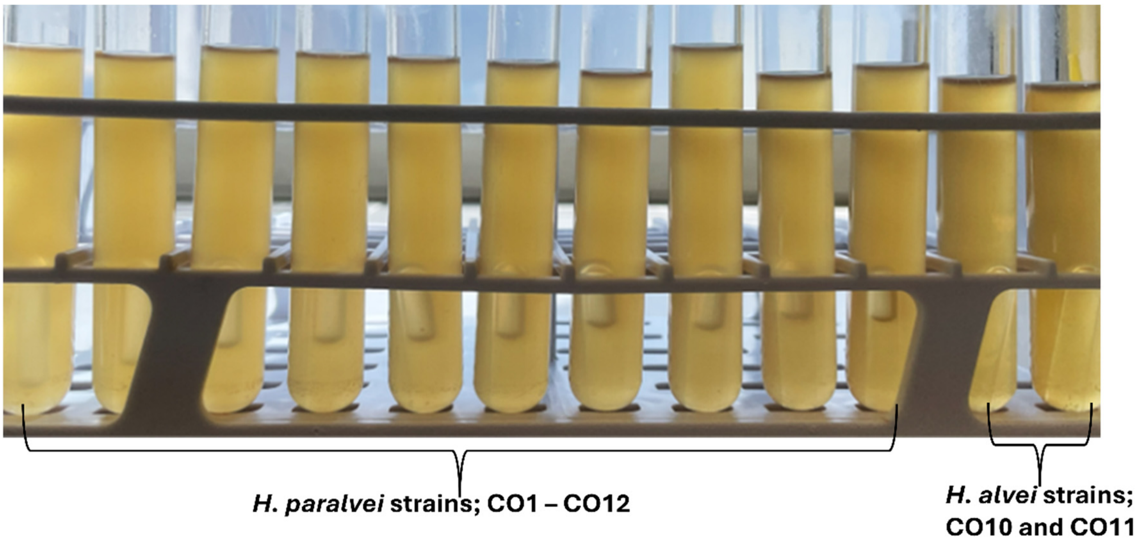

Figure S6: *Hafnia* gas production.
Gas production of *Hafnia* strains in LM17 broth after 24 hours incubation. All 10 *H. paralvei* strains produced gas within 24 hours of incubation whereas *H. alvei* strains CO10 and CO11 did not produce gas at the same time frame as others, but they were observed to produce gas following another 24 hours of incubation at Room Temperature (RT ~20 °C).

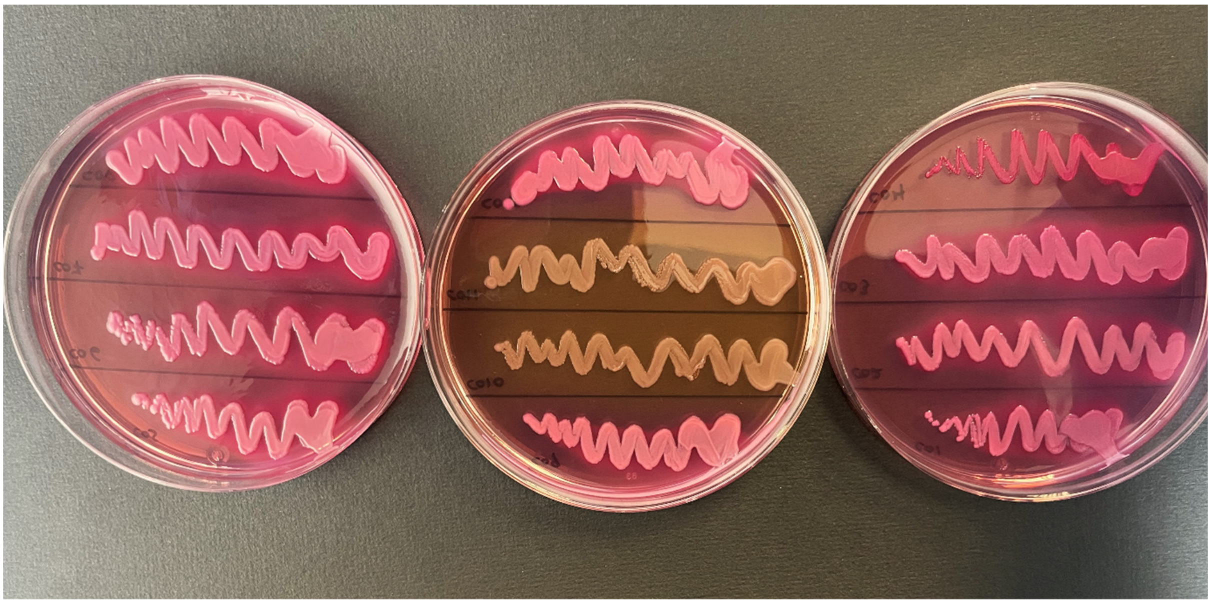

Figure S7: *Hafnia* lactose metabolism.

Lactose utilization by *Hafnia* isolates on MacConkey agar. All ten *H. paralvei* species metabolize lactose. Their colonies turned pink whereas *H. alvei* species remained yellow, indicating they do not use lactose.

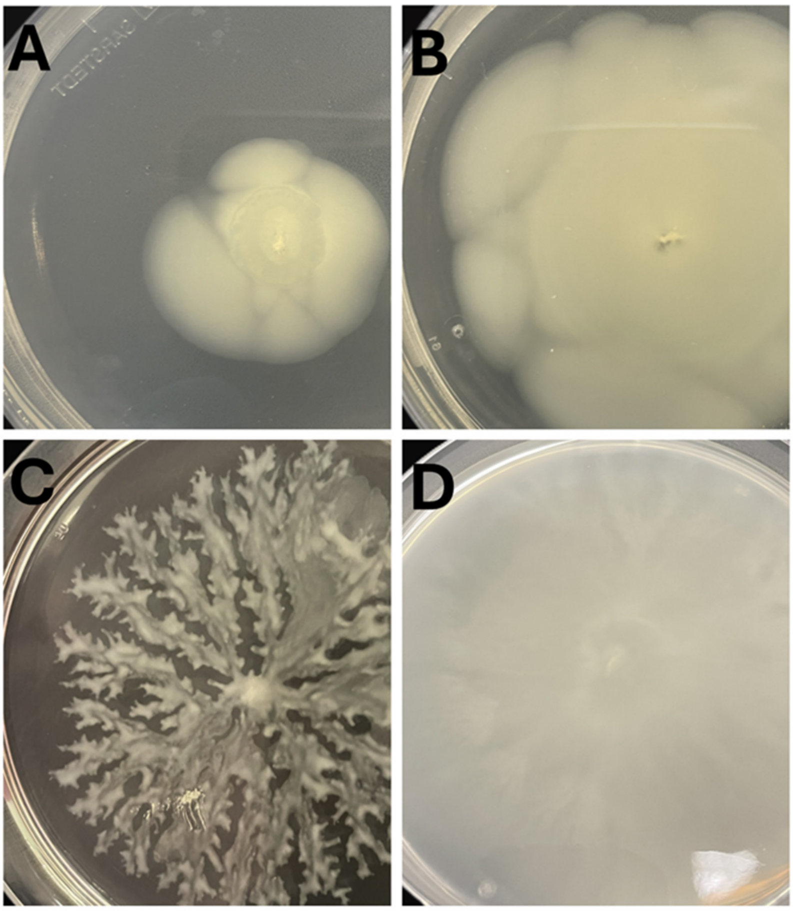

Figure S8: *Hafnia* motility assays.
Representative images of three *H. paralvei* strains (CO1, CO2 , CO3) following incubation in motility assays. Panels A and B (48 hours) display strain CO3 motility on LB (panel A) and LM17 (panel B) agar. Panels C (swarming motility) and D (swimming motility) display the motility profiles of strain CO2 (panel C) and strain CO1 (panel D) after 24 hours on Eiken agar. The type of motility displayed on Eiken agar; swimming (panel D) or swarming (panel C) is strain-dependent irrespective of species.

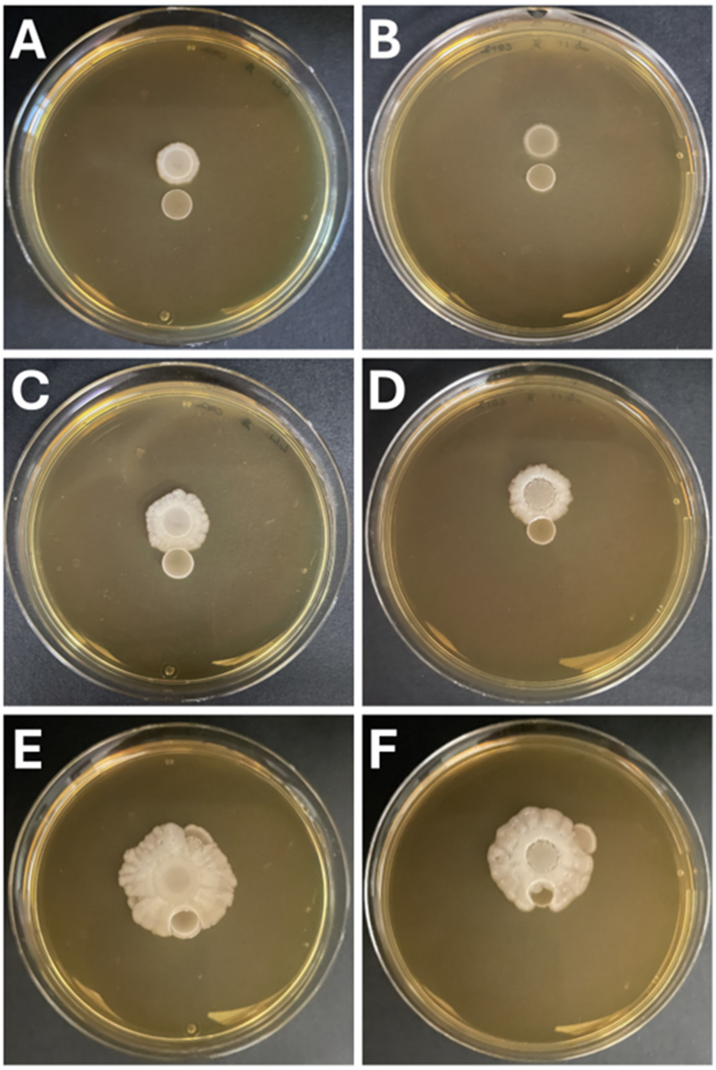

Figure S9: Interaction of *H. paralvei* CO12 isolate with LAB isolates.
Representative images of motility assays of *H. paralvei* CO12 in proximity to *L. lactis* and *S. thermophilus* isolates grown on LM17 agar. Panels (A&B) show isolates of CO12 (*H. paralvei* – top isolates) and LL1 (*L. lactis* – bottom isolate figure A) and COSt11 (*S. thermophilus* – bottom figure B*)* after 24 hours of incubation. Panels C&D show the same isolates in the same order following 72 hours of incubation at room temperature with CO12 growing towards the LAB isolates and panels E&F show figures of the isolates after six days of incubation at room temperature (RT ~20 °C) and where the *Hafnia* isolates engulf the LAB isolates.
